# Acceptability, appropriateness, and feasibility of an online facilitation training program designed to support the implementation of person-centered care in Swedish healthcare—a qualitative study

**DOI:** 10.1186/s43058-025-00752-7

**Published:** 2025-05-30

**Authors:** Ewa Carlsson Lalloo, Anna Bergström, Leif Eriksson, Lars Wallin, Emmelie Barenfeld

**Affiliations:** 1 Faculty of Caring Science, Work Life, and Social Welfare, University of Borås, Borås, Sweden; 2 Centre for Person-Centred Care (GPCC), University of Gothenburg, Gothenburg, Sweden; 3 Center for Epidemiology and Community Medicine, Region Stockholm, Stockholm, Sweden; 4 Department of Women’s and Children’s Health, Uppsala University, Uppsala, Sweden; 5 Department of Learning, Informatics, Management and Ethics, Karolinska Institutet, Stockholm, Sweden; 6 Department for Health and Welfare, Dalarna University, Falun, Sweden; 7 Institute of Neuroscience and Physiology, Department of Health and Rehabilitation, University of Gothenburg, Gothenburg, Sweden

**Keywords:** Implementation science, Implementation outcomes, Person-centered care, Patient-centered care, Facilitation, Distance education, Health personnel

## Abstract

**Background:**

Despite legislative support, PCC is not systematically practiced. An online facilitation training program targeting healthcare staff was developed in Sweden. This study aims to explore the acceptability, appropriateness, and feasibility of this facilitation training program, designed to support PCC implementation.

**Methods:**

This interview study evaluates the FaciLitating Implementation of Person-centered care (FLIP) training program according to the implementation outcomes acceptability, appropriateness, and feasibility, using deductive qualitative content analysis. FLIP integrates the Building Implementation Capacity for Facilitation (BIC-F), which focuses on behavioral change, and PCC principles. FLIP included workshops and supervision sessions held online, led by external facilitators. Between these meetings, the FLIP participants worked with implementation plans in co-creation with their colleagues. Five healthcare units, with different healthcare contexts, in Sweden, participated over 12 weeks with two healthcare staff assigned the role as internal facilitators per unit, selected and supported by their managers. All internal facilitators, managers, and external facilitators were invited to participate in evaluating FLIP. A total of 17 participants, eight internal facilitators, five managers, and four external facilitators were interviewed in semi-structured individual and group interviews.

**Results:**

FLIP was generally accepted among all participants, due to its emphasis on PCC, comprehensive content, and clear structure, as well as its blend of training, collaboration, and mutual support. Nevertheless, the acceptability was negatively affected by low attendance, low engagement due to the online format, and initial struggles with the systematic implementation model. The systematic implementation model used in FLIP was perceived as appropriate for implementing PCC in clinical practice; however, the training on PCC was viewed as insufficient, leading to challenges operationalizing PCC elements. The participants’ perceptions of FLIP’s feasibility varied; while delivery was manageable, busy schedules and technical disruptions negatively affected attendance and engagement.

**Conclusions:**

Becoming a facilitator capable of supporting the implementation of PCC is demanding and requires an understanding of both implementation and PCC. The BIC-F model was found to be accepted and appropriate, but operationalizing PCC requires more focus. Managerial support is needed to increase feasibility. Further research is required to evaluate whether facilitation skills can be trained online for large-scale PCC implementation.

**Supplementary Information:**

The online version contains supplementary material available at 10.1186/s43058-025-00752-7.

Contributions to the literature

- A novel online training program is described, aiming to combine facilitation and person-centered principles.
- The relevance of promoting PCC was seen as self-evident, resulting in overall high acceptability of the online training program among participants.
- The systematic implementation model applied in the program was perceived as appropriate for implementing PCC in clinical practice. However, the educational content on PCC was insufficient.
- The online format limited interactive and reflective learning processes, which are known to be important for operationalizing PCC.
- Overall feasibility was reduced by low attendance, as balancing professional duties with participation was challenging for internal facilitators.

## Background

Person-centered care (PCC) is defined as an ethical care model aimed at enhancing conditions for good and equal health, advocating for collaborative partnerships between patients and healthcare staff, and improving patients’ involvement in their own care process [1, 2]. Consequently, healthcare systems worldwide are becoming more sustainable by harnessing the lived experiences of patients and healthcare staff [3, 4]. This has signified a notable shift in Sweden, as well as in other European countries, toward PCC [5], reinforced by legislation and policy directives [6]. Despite these ongoing efforts, PCC is not yet systematically applied within healthcare systems [7]. Interventions aiming to implement PCC in clinical practice are therefore crucial for transforming healthcare to deliver high-quality and equitable care.

PCC can be considered a complex intervention [8], as it requires a change in managers’ and healthcare professionals’ knowledge, attitudes, and behaviors, in addition to a change in organizational culture [9]. Hence, a person-centered approach must be present at all levels of the organization [10]. Perceptions of how PCC can be implemented are influenced by factors such as its ethical foundations, operationalization in practice, and each provider’s understanding and valuation of PCC [11]. As there is no universal definition of PCC [12], it is necessary to operationalize PCC into practical activities across different areas of care to successfully implement PCC [13]. In this study, PCC is applied and operationalized based on an ethical framework developed by the University of Gothenburg Centre for Person-Centred Care (GPCC) [14]. This framework emphasizes patients as capable and resourceful people who have expert knowledge regarding their lives, goals, and motivations [15], and patients should be essential partners in their own care processes, as emphasized in the European standard CEN/TC 450 entitled “Patient involvement in healthcare—Minimum requirements for person-centred care” [16]. The GPCC framework constitutes three routines to operationalize PCC: 1) initiating the partnership between the patient and the provider by acquiring the patient’s narrative and experience of illness; 2) establishing the partnership between the patient and the provider through information sharing, deliberation, and agreeing on goals; and 3) safeguarding the partnership between the patient and the provider through documentation of the narrative and agreements [15]. Healthcare interventions based on the GPCC framework have been found to improve a range of healthcare outcomes, including care quality, self-efficacy, and experienced health [17], as well as having the potential to reduce healthcare costs [18, 19]. Educational initiatives aimed at translating PCC into healthcare practices indicate a need to enhance knowledge and understanding of PCC [20] and develop skills that effectively support the implementation of PCC [21]. Thus, there is a need for training programs that include implementation skills to facilitate PCC practices.

Facilitation has been shown to be helpful in successfully tackling the challenges that typically accompany the implementation of new ways of working in healthcare settings [22, 23]. It streamlines the change process, aligns what will be implemented with existing practices, and simplifies complexity [24]. Thus, facilitation is considered a suitable strategy to support the implementation of PCC [25, 26] and is central in the integrated Promoting Action on Research Implementation in Health Services (i-PARIHS) framework [27]. The framework proposes a dynamic interaction between the innovation to be implemented, the recipients who will utilize or be targeted by the innovation, and the context in which the innovation is implemented and facilitation has been proposed as the pivotal strategy for integrating these components [28]. It requires a facilitator—that is, an individual with a designated role in supporting the implementation process [27]. It is common to use staff as facilitators, as staff’s frontline insights can lead to more practical solutions and foster a culture of innovation and teamwork [29]. However, healthcare staff engaged in improving healthcare must be prepared to facilitate change [30] and need knowledge and implementation skills to ensure that the facilitation is effective, such as knowing how to navigate challenges [31]. Another key to a successful change of practice in an implementation process is having support from management [32–34]. Therefore, combining the training of healthcare staff in facilitation and PCC, with support from their managers, can be an effective strategy for implementing PCC in daily routines in clinical practice [26, 35]. The FaciLitating Implementation of Person-centered care (FLIP) online training package was developed to support healthcare units in implementing PCC in Sweden. Healthcare staff were assigned the role of facilitators to support the implementation of PCC with support from their managers. Hence, this study aims to explore the acceptability, appropriateness and feasibility of this online facilitation training program designed to support PCC implementation.

## Methods

### Design

This qualitative interview study deductively evaluates the FLIP training program according to the implementation outcomes of acceptability, appropriateness, and feasibility [36]. The study is reported according to the Template for Intervention Description and Replication (TIDieR) [37] (Supplementary file 1) and the Consolidated Criteria for Reporting Qualitative Research (COREQ) [38] (Supplementary file 2).

### The development of the FLIP training program

The development of the FLIP training program included the use of adaptive reflection [39], whereby experts in facilitating and implementing PCC in various health organizational levels, such as micro and meso levels, and academic organizations in Sweden, were invited to a co-creation workshop to identify the knowledge and skills facilitators need to implement PCC. The findings from the workshop were later developed employing Bloom’s taxonomy [40] and again discussed with the experts. The co-creation workshop resulted in the development of the following learning objectives for the FLIP training:

- Explain what PCC is and its purpose and provide good examples of its practice.
- Identify the organizational levels, particularly at the micro and meso levels, within the participants´ healthcare organizations and the potential impact this has on implementing PCC in their healthcare units.
- In collaboration with the manager, identify and engage different professions in implementing PCC.
- Apply a systematic implementation model to support the implementation of PCC.

The FLIP training program takes as its starting point the Building Implementation Capacity for Facilitation (BIC-F), designed and provided by the Unit of Implementation and Evaluation at the Center of Epidemiology and Community Medicine in Region Stockholm, Sweden [41]. The BIC-F is provided to individuals representing a public organization who have a facilitating function and an ongoing or upcoming implementation project they could work on during the training. The four core components of BIC-F are lectures and exercises to increase the participants’ knowledge of and skills in implementation. The first core component focuses on participants learning to apply a systematic implementation model to support behavioral change processes [42]. This model includes six steps: (i) Describe the overall goal of the implementation; (ii) identify and specify target behavior(s); (iii) for each behavior, analyze what is needed for behavioral change to occur; (iv) choose implementation strategies based on the analysis in the third step; (v) apply implementation strategies; and (vi) monitor the occurrence of the target behavior. Participants also learn how to ensure the sustainability of the implementation through follow-up, adaptation, and alignment. The second core component consists of lectures and exercises to increase participants’ knowledge of and skills in facilitation. Core component three is about working on the implementation plan in between workshops and co-creating it with relevant organizational stakeholders by anchoring it with them. Lastly, core component four entails peer support and feedback on performance from workshop leaders. Participants are also encouraged to interact with each other and with course leaders throughout the workshops. To target the FLIP learning objectives, the BIC-F training was complemented with educational content on PCC, with person-centered principles being integrated with the four core components. The GPCC model [14] and the European standard CEN/TC 450 defining the minimum requirements for PCC [16] served as a basis for the educational content on PCC.

### The delivery of the FLIP training program

In total, the FLIP training program included seven online half-day workshops and two 30-min online supervision sessions with each healthcare unit, provided over 12 weeks between October 2022 and February 2023 (Table 1). Since the workshops and supervision sessions in the training program were conducted through online meetings, it utilized blended learning techniques to enhance learning activities, including reflections, discussions, videos, and various exercises. Moreover, learning activities were carried out at units between the online sessions.

**Table 1:** Overview of the content and structure of FLIP

Session	Activity and content	Participants
Workshop 1	- Lecture: Introduction to person-centered care; Implementation; A systematic implementation model; Facilitation - Exercise: Brainstorming on facilitation skills and attributes (as one group)	- Internal facilitators - Managers
In-between workshops	- Home assignment: Initiate implementation plan	- Internal facilitators - Healthcare unit colleagues - Managers
Workshop 2	- Lecture: Person-centered care	- Internal facilitators
In between workshops	- Continue developing implementation plan - Reflect on relevant actors in your implementation	- Internal facilitators - Healthcare unit colleagues - Managers
Workshop 3	- Exercise: Applying the systematic implementation model to fictitious case - Lecture and exercise: Stakeholder analysis - Exercise: Identify target behaviour in PCC	- Internal facilitators
In between workshops	- Continue developing implementation plan - Conduct a stakeholder analysis	- Internal facilitators - Healthcare unit colleagues - Managers
Workshop 4	- Lectures: Applying the systematic implementation model; Implementation in an organization; Person-centered care - Group supervision (3–4 participants): Reflection on participants’ application of the systematic implementation model on own case	- Internal facilitators
In between workshops	- Home assignment: Anchoring the implementation plan with key stakeholders and revise plan according to feedback from stakeholders - Individual supervision session entailing discussion implementation plan and other topics relevant to the facilitator (30 min)	- Internal facilitators - Healthcare unit colleagues - Managers
Workshop 5	- Lectures: Communication; Applying the systematic implementation model; Providing feedback - Exercise: training on how to communicate about the implementation case using the elevator pitch technique - Group supervision (3–4 participants): Reflection on participants’ application of the systematic implementation model on own case	- Internal facilitators - Guest lecturer
In between workshops	- Home assignment: Anchoring the implementation plan with key stakeholders and revise plan according to feedback from stakeholders - Individual supervision sessions entailing discussion implementation plan and other topics relevant to the facilitator (30 min)	- Internal facilitators - Healthcare unit colleagues - Managers
Workshop 6	- Lecture: Change resistance - Exercise: Applying facilitation tools relevant for change resistance and role-play on change resistance	- Internal facilitators - Guest lecturer
In between workshops	- Home assignment: Revisions of implementation plan	- Internal facilitators - Healthcare unit colleagues - Managers
Workshop 7	- Lecture: Sustaining implementation through follow-up; Adaptation; Alignment - Exercise: Applying the systematic implementation model to fictitious case (3–4 participants) - Reflections on lessons learned (as one group)	- Internal facilitators

The FLIP training program was led by two external facilitators (EFs) with expertise in implementation science, in collaboration with two EFs with expertise in PCC. A guest lecturer with expertise in communication and change resistance also delivered two lectures online. The external facilitators and guest lecturer had no connection with the healthcare units and were not involved in clinical work. The EFs role was to deliver the FLIP training online to the participating healthcare units and equip the IFs with the necessary skills to independently lead the implementation at their respective healthcare unit. The EFs led the workshops and provided supervision sessions with each healthcare unit in online meetings. These sessions focused on collaborating on the implementation plan, during which the EFs supported the IFs and managers for each healthcare unit, enabling them to determine the necessary actions to implement PCC at their specific unit.

The IFs were expected to attend all online workshops and supervision sessions and, in between, create an implementation plan for driving the healthcare unit’s internal change process toward increased person-centeredness. The implementation plan was created collaboratively with their colleagues. The managers participated in the first workshop and the two supervision sessions to understand FLIP and to effectively support their IFs in driving the change process.

### Recruitment and study participants

The project was announced through a network established by the GPCC, and a contact person from Southern Sweden emailed GPCC, stating that five healthcare units in the region were interested in participating. All five units were invited and agreed to participate in the research project. Hence, there was no established relationship between the participants and the authors prior to the study’s commencement.

The five units were all located in a small sized city in southern Sweden. The healthcare units represented both primary and specialized, hospital, care, and targeted a variety of patients concerning main diagnoses, ages and acute vs chronic conditions. The units had varying experiences of PCC, ranging from none to having completed self-driven digital education on PCC. At the study baseline, the number of healthcare staff employed at each unit ranged between 34–69 individuals (including staff on maternity leave or sick leave). The categories of healthcare staff employed at the different units varied based on the specialty and organization of each healthcare unit; however, the majority were nurses or nursing assistants. Two staff members from each healthcare unit were selected by their unit manager to serve as internal facilitators (IFs) in FLIP. The managers were not required to explain their selection of IFs;, however, factors such as the selection of staff members with prior improvement work experience in the unit or those who showed an interest in PCC were mentioned. To stimulate the establishment of a coalition between the two IFs and their manager, the managers of each healthcare unit were invited to attend the first workshop and the two supervision sessions.

All IFs (*n* = 10) and managers (*n* = 6) who participated in the FLIP training program were offered the opportunity to participate in its evaluation at the end of the program, alongside the EFs (*n* = 4). Two IFs and one manager did not participate in the interviews. A total of 17 individuals were included in the analysis: the IFs (*n* = 8), their managers (*n* = 5), and the EFs (*n* = 4). All IFs were women and worked as physicians, registered and specialized nurses, or registered nursing assistants. The managers were all heads of units, and most were women. The EFs had different academic competencies; all had healthcare professional education, and half were men.

### Data collection

Data on attendance levels in FLIP were registered by the EFs. Semi-structured individual and group interviews were conducted in February 2023 after the end of the program. Interviews were conducted both on-site at the participants’ workplace and online via Zoom (Table 2). ECL conducted the interviews; in the digital group interviews, LW contributed with follow-up questions. No field notes were taken. Neither ECL nor LW were involved in delivering the FLIP program. A semi-structured interview guide focusing on FLIP’s acceptability, appropriateness, and feasibility, with open follow-up questions, was developed and used (Supplementary file 3). No pilot testing was performed. The guide was based on Proctor et al.’s [36] definition of the implementation outcomes of acceptability, appropriateness, and feasibility. When developing the questions, the items from Weiner’s scale were also used to assess these implementation outcomes [43]. All interviews were audio-recorded and transcribed verbatim. The transcripts were not returned to the participants.

**Table 2:** Overview of healthcare unit representation, data collection methods, and amount of data from internal facilitators, managers, and external facilitators

	**Internal facilitators** **(*n* = 8)**	**Managers** **(*n* = 5)**	**External facilitators** **(*n* = 4)**
Number of represented healthcare units	5	4	NA
Number of group interviews Number of individual interviews	3 1	1 2	1 0
Number of digital interviews Number of on-site interviews	2 2	1 2	1 NA
Length in minutes: range; mean	23–78; 55	38–59; 47	88

### Data analysis

The attendance at workshops and supervision sessions was recorded and compared to the maximum potential attendance. The transcribed interviews were analyzed using qualitative deductive content analysis [44]. First, all interviews were read to understand and gain a sense of the whole content. Next, we focused on identifying meaning units in each interview and categorizing these meaning units according to the three implementation outcomes of acceptability, appropriateness, and feasibility, as described by Proctor et al. [36] (see Table 3). All meaning units corresponding to each implementation outcome were clustered separately for IFs, managers, and EFs. ECL and EB primarily performed the analysis. Descriptions of the raw data underpinning each category were verified by LW, LE, and AB. Meaning units that overlapped between two categories were discussed by the entire group until agreement on their categorization was reached.

**Table 3:** The definitions of acceptability, appropriateness, and feasibility used in the deductive analysis to evaluate the FLIP program, based on Proctor et al. [36]

Implementation outcome	Study-specific definition
Acceptability	The perception among the study participants regarding whether FLIP was agreeable, palatable, or satisfactory
Appropriateness	The perceived fit, relevance, or compatibility of FLIP for the healthcare units and the perceived fit of FLIP for supporting the implementation of PCC by training healthcare staff in the role of facilitators
Feasibility	The extent to which FLIP could be successfully used or carried out within their healthcare units

The psychometrically validated scale developed by Weiner et al. [37] was used to clarify the implementation outcomes, helping the research team to further refine and delimit the three implementation outcomes in the analysis.

### Ethical considerations

This study was reviewed by the Swedish Ethical Review Authority and was determined to not need ethical approval (Ref. no: 2022–03836-01). Nevertheless, the study followed ethical principles [45]. The participants were informed about the study and given the opportunity to ask questions. Written or oral informed consent was obtained from all study participants and documented by the researchers. Participation in the interviews was voluntary for all participants, and the participants could withdraw at any time without providing a reason.

## Results

The presentation of the results is divided into two sections: 1) FLIP attendance and 2) perceptions on the implementation outcomes of FLIP’s acceptability, appropriateness, and feasibility.

### FLIP attendance

The overall attendance among the participants of FLIP varied. Table 4 illustrates the attendance per healthcare unit throughout the FLIP training program.

**Table 4:** Number of attending individuals at different workshops and supervision sessions per healthcare unit

	Available individuals	WS 1	WS 2	WS 3	WS 4	WS 5	WS 6	WS 7	SV 1	SV 2
Healthcare unit 1	IF (*n* = 2)	2	2	1	2	2	0	2	2	2
	Manager^a^(*n* = 1 + 1)	1							2	0
Healthcare unit 2	IF^b^(*n* = 2)	2	1	2	2	1	2	2	0	1
	Manager (*n* = 1)	1							0	1
Healthcare unit 3	IF (*n* = 2)	2	1	0	2	0	1	0	0	2
	Manager (*n* = 1)	1							0	1
Healthcare unit 4	IF (*n* = 2)	2	1	0	2	1	1	0	2	2
	Manager (*n* = 1)	1							1	0
Healthcare unit 5	IF (*n* = 2)	2	2	2	2	2	2	1	2	2
	Manager (*n* = 1)	0							0	0

*WS* workshop, *SV* supervision

^a^Unit 1 changed manager; thus, two managers attended SV 1

^b^Unit 2 changed an IF after WS 1

### Perceptions on implementation outcomes

#### Acceptability

FLIP was well accepted by the participants for its comprehensive content and its combination of PCC and implementation training, with particularly high approval for the facilitation and implementation components. PCC was viewed as a valuable part of healthcare, contributing to FLIP’s acceptability. Additionally, the IFs viewed participation in FLIP as an opportunity for professional growth. The managers particularly appreciated FLIP for enhancing the IFs’ problem-solving skills and fostering innovation in healthcare beyond just PCC.

FLIP’s diverse learning activities, such as lectures, practical exercises, and video material, enhanced its acceptability. While some participants initially struggled with understanding the systematic implementation model and found the language theoretical and complex, their acceptance grew as they engaged with FLIP’s straightforward, step-by-step structure, as presented in the workshops.

Exercises such as brainstorming helped participants understand the challenge of achieving a common understanding, especially regarding PCC. Facilitators in healthcare units viewed these exercises as applicable to promoting staff reflections on PCC. A key exercise involved applying the systematic implementation model to a fictional case, which helped IFs to understand the model and consider practical PCC implementation. Thus, participants desired more examples of PCC in clinical practice.

We are raised with TVs and computers. We need to be entertained. Most things that catch my interest must contain a certain drama: some real, some horror, and some sunshine examples. There must be something that you can tie to reality. The best thing I know when I’m learning something new is to hear someone sharing their experience. (IF)

The opportunities for collaboration and joint learning during the workshops—particularly in smaller discussion groups—strengthened FLIP’s acceptability among the IFs. The collaboration activities with their own colleagues at their healthcare units varied, but the activities initiated by the EFs fostered the exchange of experiences, bringing new perspectives and enriched understanding of PCC implementation. Mutual support between FLIP participants and EFs helped the participants navigate uncertainties and achieve the learning objectives. All participants, including EFs, highly valued supervision sessions as these sessions enhanced the participants’ understanding of the implementation model and PCC, while also serving as motivational checkpoints, especially for those who missed sessions.

There have been really great things that emerged (during the supervision sessions), which are truly relevant for person-centered care and the changes one wants to make. (EF)

Missing sessions decreased acceptability and affected the IFs’ engagement and ability to plan implementation activities as part of their home assignments in between the workshops. As a result, some IFs felt unprepared for the workshops and implementation work in between sessions, leading to feelings of guilt and pressure. For those who missed sessions or did not work on the assignments between sessions, FLIP became more of a burden than a supportive program.

When you have the chance and opportunity to do this, you want to do it properly. The motivation disappeared when it became pressure. (IF)

The acceptability of FLIP’s online format varied. Managers found the online format convenient, making it easier for them to participate in FLIP, whereas many of the IFs said that they would have preferred in-person meetings.

In summary, FLIP was found to be acceptable due to its comprehensive content of PCC and facilitation, clear structure, a blend of learning activities, joint learning in workshops, and support from the EFs. However, acceptability was negatively influencedby initial struggles with the systematic implementation model, challenges in attending sessions, and the online format.

#### Appropriateness

Participants’ perceptions of FLIP’s appropriateness varied. Overall, FLIP was seen as highly appropriate and valuable as a tool for supporting the implementation of PCC in clinical practice. Benefits such as increased patient involvement, improved safety, and a more sustainable work environment reinforced this view. However, FLIP’s appropriateness was also questioned, as the FLIP participants felt it did not provide enough knowledge about PCC, although FLIP did offer a deeper understanding of PCC. The participants also described difficulties in implementing person-centered principles, as they already applied PCC in their current practices. EFs also doubted FLIP’s suitability for supporting PCC implementation, noting that FLIP might need more PCC content and that reflections about PCC during the sessions were uneven and sometimes lacking in depth. The online format was also perceived to limit spontaneous interactions and real-time reflections during the workshops, and EFs expressed concerns about its impact on learning.

FLIP’s appropriateness was perceived as high because the systematic implementation model in FLIP made PCC more tangible and concrete. Healthcare units that developed PCC implementation plans found them helpful in facilitating PCC. However, not all participants found the systematic implementation model universally appropriate. Its complexity could pose challenges, leading to confusion about expected outcomes, especially when an implementation could be considered complete or successful. Challenges also emerged when participants missed workshops or supervision sessions, resulting in a lack of understanding of the steps in the implementation model. Recorded lectures for those who missed workshops were suggested, as the materials provided were seen as insufficient for catching up. EFs pinpointed this challenge:

The big challenge has been... It’s a series of sessions built on each other, and the foundation, this systematic implementation model, is based on working step by step.//It’s like starting to learn a math problem, if you haven’t understood foundation A, it becomes quite difficult to follow foundation B. (EF)

Moreover, EFs raised concerns about whether FLIP adequately prepared healthcare staff for the demanding role of facilitators. To increase the chances of success in FLIP, at least one manager described choosing IFs likely to possess the potential to meet the challenges of participating in FLIP and implementing PCC. The IFs in daily clinical practice struggled to prioritize FLIP alongside their care duties, and some felt uneasy about leaving colleagues to manage their clinical responsibilities when participating in workshops, making consistent engagement difficult.

There were three people absent that day. You can’t just sit down at a computer and let the others run themselves ragged. (IF)

In summary, FLIP was considered appropriate, specifically regarding incorporating the systematic implementation model. FLIP was also perceived as less appropriate due to challenges applying PCC elements and concerns about whether it adequately prepared staff for their facilitator roles.

#### Feasibility

Perceived feasibility varied among the participants and was negatively impacted by low attendance levels. EFs found FLIP’s online delivery feasible but described challenges regarding participant continuity and low attendance (see Table 4).

All FLIP participants reported that FLIP’s feasibility relied on careful planning to ensure attendance and engagement during and between sessions. EFs observed that the heavy workload and the demands of clinical duties made it challenging for IFs to prioritize the training program. EFs also noted that IFs were occasionally reserved during the workshops, which they assumed was because of differing expectations of FLIP as an educational program. Some participants appeared unprepared for the workshops’ interactive format, having anticipated a lecture-based approach.

IFs and managers reported lower-than-expected participation and engagement during and between workshops. They said the FLIP program coincided with an exceptionally busy period at their units. Factors such as vacations, other educational commitments, and high levels of staff sick leave, which were described as even more challenging than during the COVID-19 pandemic, affected the attendance level.

Between two workshops where I hadn’t even had time to think about PCC once, we all agreed that we hadn’t even had time to think about implementation.//There had been chaos at the hospital. So, it felt tough to join a workshop, knowing that I had not worked with the implementation plan. But then it turned out that no one had. (IF)

The online format further constrained the opportunities for reflection. The online format was considered convenient, but attending FLIP from workstation computers frequently resulted in disruptions caused by technical issues, such as problems logging in, and interruptions from colleagues. It also led to the FLIP participants being grouped in their units during discussions, which made it challenging to exchange experiences on PCC between units and thus limited the opportunity to receive new input from peers. To improve attendance rates, it was suggested that sessions should be condensed into fewer days, as IFs found it more manageable when they could dedicate entire days to the FLIP program. Another recommendation was to participate in the online workshops off-site, either alone or with the other facilitator, to minimize distractions and maintain focus.

IFs noted that the program’s feasibility improved when their managers created supportive conditions, such as allocating dedicated time or facilitating reflective discussions. These conditions helped IFs participate fully and achieve FLIP’s learning objectives.

They had a week where both of them (the facilitators) could work on it. And then they got started. After that, they were super enthusiastic.//They wanted to do it, but they were just quite exhausted, having missed sessions. (Manager)

The conditions given and the support provided by the manager varied between the healthcare units, as did the collaboration between the managers and IFs. Some managers regularly discussed PCC implementation with their IFs, while others supported it by emphasizing its importance to staff, such as by giving IFs time to present at meetings. Some managers noted that the implementation of PCC was now driven by the healthcare team, in contrast to previous top-down implementation projects.

I think it [the implementation] grows from the ground. And it feels... you know, it makes you feel a bit satisfied when you listen because it means that it’s not something we impose from above, but rather it comes from the bottom up. (Manager)

In summary, the feasibility of FLIP varied. While delivery was manageable, low attendance and engagement, busy schedules, and technical disruptions affected overall participation and engagement.

## Discussion

This study evaluated the acceptability, appropriateness, and feasibility of an online program training healthcare staff to take on the role of facilitators in supporting the implementation of PCC within their healthcare units. Our results demonstrate that the participants accepted the educational content of FLIP due to its blend of PCC and facilitation training. These contents were considered highly relevant for improving care and fostering professional growth. The training of facilitators in line with BIC-F has been shown to increase knowledge and skills, helping facilitators appreciate the value of a systematic implementation model in their work [41]. Our results indicate that learning to apply the systematic implementation model (core component 1) was highly acceptable, especially for those with high attendance levels. It can thus be assumed that BIC-F can bridge the gap between theory and practice by supporting the implementation of PCC.

The results in this study show that some IFs had difficulty defining person-centered actions beyond their current practice and thus questioned the appropriateness and feasibility of FLIP in applying the BIC-F model to implement PCC. A known challenge in the implementation of PCC is that it is not only about changing what staff *do* (i.e., their “doing”) but also about changing how the staff *are* in relation to others (i.e., their “being”) [46]. To effectively implement PCC, training targeting staff, facilitators, or leaders should address the support of both translating PCC into observable and practical PCC actions and fostering clinical mindsets, such as attitudes and relational skills [35, 47]. Becoming a PCC facilitator is highly relational [25, 35, 48], so program components strengthening facilitators’ person-centered “being” are required. The BIC-F model’s focus on supporting behavioral change [49, 50] led FLIP to emphasize practical actions. This emphasis may have resulted in insufficient attention on fostering work in partnership, suggesting that the integration of relational components—as used in other facilitation training programs [35, 51]—requires strengthening in FLIP.

Our results show that one of the most appreciated parts of FLIP was working together with other FLIP participants and EFs to define and implement PCC. These activities, which the EFs led, helped the participants collaboratively discuss and reflect on how to translate PCC into practical applications. A systematic review [52] reporting on education of various health professions showed that reflection is regarded as essential for advancing professional competence, bridging theory and practice, fostering continuous learning, and increasing empathy. Moreover, critical reflectivity is advocated in training programs to support PCC to increase self-awareness, along with assignments challenging current norms [26]. It is also assumed that time and continuous reflection are needed for a facilitator to discover new nuances of person-centeredness [53]. Therefore, in future, the FLIP training program should emphasize both individual and collaborative reflection regarding the principles of PCC and the process of becoming a person-centered facilitator.

Our results revealed that holding meetings online – without face-to-face interaction – was a a challenging aspect of FLIP. Online meetings sometimes hindered group reflections and shared experiences on PCC across units, as the participants struggled to engage in real-time discussions and exchange ideas naturally. A hybrid approach to combine online learning with in-person interactions, in a way that many of the discussions could occur face-to-face, might have been an option in FLIP. Additionally, learning activities such as reflections in breakout rooms, which have worked well in other online training programs [54], could also be an option. However, such an approach was judged to be infeasible, as the participants faced challenges such as limited access to computers and working in environments with frequent interruptions. To address these issues, the IFs suggested rescheduling FLIP into whole-day in-person sessions, and to physically leave the healthcare unit.

The FLIP program was perceived as appropriate, applicable, and feasible for some participants, but not for others. A key factor influencing these perceptions was the relatively low attendance in workshops and supervision sessions, which disrupted the sequential structure of FLIP; as a result, FLIP’s appropriateness to train staff into facilitators of PCC was questioned. In line with a systematic review [55] of staff-reported barriers and facilitators to implementation processes, our results showed that the care of patients was prioritized over the implementation work in some units due to stress and time-management challenges. This result indicates that the training was viewed as an add-on activity instead of being integrated into ordinary practice as expected. Implementing PCC requires significant time and resources and needs to be planned for [19]. Consistent with previous research [56], our finding emphasizes that the staff members being trained as facilitators need their managers’ support in effectively planning their time and engaging collaboratively in implementation and practice change. To better support this process, mandatory participation of managers in FLIP is essential to ensure that they are actively involved in fostering commitment, negotiating conditions, and maintaining the changes throughout the implementation [26]. Novice and experienced facilitators are known to face various challenges and to need different types of support in developing person-centered facilitation skills [35], and this was also evident in our study. As FLIP is aimed at healthcare staff, who can be considered novice facilitators, it is necessary to consider such challenges when selecting staff for the role and to prepare the staff for the work that will be needed, while ensuring supportive structures.

High acceptability of training programs such as FLIP is crucial for the successful uptake and sustainability of PCC, as acceptability is often linked to greater engagement and adherence [36]. Therefore, the results of this study imply that more precise communication regarding the goals and vision of FLIP and the implementation of PCC in clinical practice is needed. This would include emphasizing patient involvement and incorporating patient narratives into FLIP. In addition, greater emphasis should be placed on core component 3, i.e., working on the implementation plan by making it an active, ongoing, and mandatory process. Participants should treat the plan as a living document, continuously engaging with and refining it throughout the training.

### Strengths and limitations

A deductive analysis is suitable for seeking a contextualized understanding of a topic with limited existing knowledge [44]. This study evaluated FLIP using Proctor’s implementation outcomes framework [36], which focuses on acceptability, appropriateness, and feasibility. Conducting a qualitative analysis posed challenges, as the implementation outcomes overlapped and sometimes involved inconsistent terms, resulting in potential semantic similarities in which meaning units could apply to more than one category. To strengthen credibility and ensure that the categories were conceptually and empirically grounded, ECL and EB cooperated closely during categorization, followed by discussions with AB, LE, and LW.

The low attendance levels might undermine the credibility of this study to some extent and thus affect the trustworthiness of the results, particularly those indicating that FLIP delivered limited new information about PCC. The challenges the participants experienced when leaving their clinical duties also affected the data collection. Potential barriers to digital interviews were addressed by using alternative interview techniques [57]. Instead of conducting on-site focus group discussions, the researchers had to adopt a flexible approach, offering interview opportunities tailored to the participants’ availability and preferences. This resulted in more individual interviews and a higher proportion of digital interviews. The flexible approach probably enabled a more extensive dataset, assessed as sufficient for information power [58].

A convenience sampling of healthcare units may have produced sampling bias. Additionally, the fact that all the facilitators wanted to participate in FLIP might have led to a positive result bias, limiting the trustworthiness of the results. Therefore, it is important to interpret the results while considering the specific sample and context of the study [44].

The study was conducted in real-world clinical settings, including five different healthcare units with varying contextual conditions regarding staff, target population, and previous experience of PCC. This can be considered a strength of the study, as it enhances the transferability of the results to similar healthcare contexts. By evaluating FLIP in a real-world context, this study provides valuable insights and highlights the difficulties and challenges associated with implementing change in healthcare. An additional strength of this study is that the research group included both male and female researchers from various healthcare professions and with competencies in both PCC and implementation science, increasing the trustworthiness of this work.

## Conclusion

As the FLIP training program was delivered in real-world healthcare settings, the results from our evaluation of its acceptability, appropriateness, and feasibility revealed several challenges. This study found that the BIC-F model was viewed as both highly acceptable and highly appropriate; however, its focus on the educational content and operationalization of PCC requires more attention. Feasibility was affected by various factors, including challenges attending FLIP because of a high workload. Becoming a facilitator with the ability to support the implementation of PCC in practice is demanding and requires an understanding of both implementation and PCC. Further research is necessary to evaluate the suitability of FLIP’s online format and its focus on facilitation skills in implementing PCC, particularly for large-scale applications.

## Supplementary Information

Supplementary Material 1.

Supplementary Material 2.

Supplementary Material 3.

## Data Availability

The datasets generated and/or analyzed during the current study are not publicly available, as the participants of this study did not give written consent for their data to be shared publicly. Data is available from the corresponding author upon reasonable request.

## Abbreviations

BIC-F: Building Implementation Capacity for Facilitation
COREQ: Consolidated Criteria for Reporting Qualitative Research
EF: External facilitator
FLIP: FaciLitating the Implementation of Person-centered care
GPCC: The University of Gothenburg Centre for Person-Centred Care
IF: Internal facilitator
i-PARIHS: The Integrated-Promoting Action on Research Implementation in Health Services
PCC: Person-centered care
SV: Supervision sessions
TIDieR: Template for Intervention Description and Replication
WS: Workshops

## References

[1] McCormack B, McCance TV. Development of a framework for person-centred nursing. J Adv Nurs. 2006;56(5):472–9. 17078823 10.1111/j.1365-2648.2006.04042.x

[2] Ekman I. Practising the ethics of person-centred care balancing ethical conviction and moral obligations. Nurs Philos. 2022;23(3):e12382. 10.1111/nup.12382. Epub 2022 Feb 25. PMID: 35213781; PMCID: PMC9285079. 10.1111/nup.12382 PMC9285079 35213781

[3] United Nations (UN). Transforming our world: the 2030 agenda for sustainable development. 2015. https://sdgs.un.org/publications/transforming-our-world-2030-agenda-sustainable-development-17981. Accessed 12^th^ March 2024.

[4] World Health Assembly, 69. Framework on integrated, people-centred health services: report by the Secretariat. World Health Organization. 2016. https://iris.who.int/handle/10665/252698 . Accessed 20^th^ August 2024.

[5] Rosengren K, Buttigieg SC, Badanta B, Carlstrom E. Diffusion of person-centred care within 27 European countries—interviews with managers, officials, and researchers at the micro, meso, and macro levels. J Health Organ Manag. 2022;ahead-of-print(ahead-of-print). 10.1108/JHOM-02-2022-0036. PMID: 36367331. 10.1108/JHOM-02-2022-0036 36367331

[6] Regeringskansliet- Socialdepartementet och Sveriges kommuner och regioner (SKR). God och nära vård 2023. En omställning av hälso- och sjukvården med primärvården som nav. Överenskommelse mellan staten och Sveriges Kommuner och Regioner. [Agreement between the Swedish government and the Swedish Association of Local Authorities] https://skr.se/download/18.436e32a318c8af0ae994036/1703172720013/Overenskommelse-god-nara-vard-2024.pdf. Accessed 15^th^ September 2024.

[7] Gyllensten H, Björkman I, Jakobsson Ung E, Ekman I, Jakobsson S. A national research centre for the evaluation and implementation of person-centred care: content from the first interventional studies. Health Expect. 2020;23(5):1362–75. 32808455 10.1111/hex.13120 PMC7696144

[8] Skivington K, Matthews L, Simpson SA, Craig P, Baird J, Blazeby JM, et al. A new framework for developing and evaluating complex interventions: update of Medical Research Council guidance. Br Med J (Online). 2021;374:n2061–n2061. 10.1136/bmj.n2061 PMC8482308 34593508

[9] Moore L, Britten N, Lydahl D, Naldemirci Ö, Elam M, Wolf A. Barriers and facilitators to the implementation of person-centred care in different healthcare contexts. Scand J Caring Sci. 2017;31(4):662–73. 27859459 10.1111/scs.12376 PMC5724704

[10] Backman A, Ahnlund P, Lövheim H, Edvardsson D. Nursing home managers’ descriptions of multi-level barriers to leading person-centred care: a content analysis. Int J Older People Nurs. 2024;19(1):e1258111. 10.1111/opn.12581 37859588

[11] Fridberg H, Wallin L, Tistad M. The innovation characteristics of person-centred care as perceived by healthcare professionals: an interview study employing a deductive-inductive content analysis guided by the consolidated framework for implementation research. BMC Health Serv Res. 2021;21(1):904. 34479553 10.1186/s12913-021-06942-y PMC8414852

[12] Byrne A-L, Baldwin A, Harvey C. Whose centre is it anyway? Defining person-centred care in nursing: an integrative review. PLoS ONE. 2020;15(3):e0229923–e0229923. 32155182 10.1371/journal.pone.0229923 PMC7064187

[13] McCormack B, Dewing J, McCance T. Developing person-centred care: addressing contextual challenges through practice development. Online J Issues Nurs. 2011;16(2):1–12. 22088152

[14] Ekman I, Swedberg K, Taft C, Lindseth A, Norberg A, Brink E, et al. Person-centered care—ready for prime time. Eur J Cardiovasc Nurs. 2011;10(4):248–51. 21764386 10.1016/j.ejcnurse.2011.06.008

[15] Britten N, Ekman I, Naldemirci Ö, Javinger M, Hedman H, Wolf A. Learning from Gothenburg model of person centred healthcare. Br Med J. 2020;370:m2738–m2738. 32873594 10.1136/bmj.m2738

[16] European Committee for Standardization. CEN/TC450—Patient involvement in person-centred care. EN17398:2020. 2020. https://standards.cen.eu/dyn/www/fp5204:7:0::::FSP. Accessed 10^th^ February 2024.

[17] Fors A, Ekman I, Taft C, Björkelund C, Frid K, Larsson MEH, et al. Person-centred care after acute coronary syndrome, from hospital to primary care—a randomised controlled trial. Int J Cardiol. 2015;187:693–9. 25919754 10.1016/j.ijcard.2015.03.336

[18] Swedberg K, Cawley D, Ekman I, Rogers HL, Antonic D, Behmane D, et al. Testing cost containment of future healthcare with maintained or improved quality—The COSTCARES project. Health Sci Rep. 2021;4(2):e309. 34141903 10.1002/hsr2.309 PMC8180514

[19] Gyllensten H, Tistad M, Fridberg H, Wallin L. Analysis on personnel costs and working time for implementing a more person-centred care approach: a case study with embedded units in a Swedish region. BMJ Open. 2023;13(10):e073829–e073829. 37821128 10.1136/bmjopen-2023-073829 PMC10582865

[20] Cable C, McCance T, McCormack B. Knowing, Being and Becoming a Person-Centred Nurse Leader: Findings from a Transformative Professional Development Programme. Nursing reports (Pavia, Italy). 2024;14(4):3165–77. 39449467 10.3390/nursrep14040230 PMC11503291

[21] Carlsson-Lalloo E, Temple F, Berg M, Berg U, Désiré AM, Mulunda A, Bogren M. Testing the feasibility of a translated and culturally adapted person-centred training programme in maternal and newborn healthcare in Democratic Republic of Congo: a process evaluation. Sex Reprod Healthc. 2024;40:40. 10.1016/j.srhc.2024.100979 38754346

[22] Moussa L, Garcia-Cardenas V, Benrimoj SI. Change facilitation strategies used in the implementation of innovations in healthcare practice: a systematic review. J Change Manag. 2019;19:283–301.

[23] Eriksson L, Huy TQ, Duc DM, Ekholm Selling K, Hoa DP, Thuy NT, et al. Process evaluation of a knowledge translation intervention using facilitation of local stakeholder groups to improve neonatal survival in the Quang Ninh province. Vietnam Trials. 2016;17(1):23. 26762125 10.1186/s13063-015-1141-z PMC4711002

[24] Cranley LA, Cummings GG, Profetto-McGrath J, Toth F, Estabrooks CA. Facilitation roles and characteristics associated with research use by healthcare professionals: a scoping review. BMJ Open. 2017;7(8):e014384. 28801388 10.1136/bmjopen-2016-014384 PMC5724142

[25] Dellenborg L, Wikström E, Andersson EA. Factors that may promote the learning of person-centred care: an ethnographic study of an implementation programme for healthcare professionals in a medical emergency ward in Sweden. Adv Health Sci Educ Theory Pract. 2019;24(2):353–81. 30632026 10.1007/s10459-018-09869-y PMC6483949

[26] McCormack B, Peelo-Kilroe L, Codd M, Baldie D. A strategically engaged programme of person-centred culture development in health services: the courage of the Irish! In: Innovative staff development in healthcare. Dublin, Ireland: Springer International Publishing; 2021. p. 101–114.

[27] Harvey G, Kitson A. PARIHS revisited: from heuristic to integrated framework for the successful implementation of knowledge into practice. Implement Sci. 2016;11(1):33. 27013464 10.1186/s13012-016-0398-2 PMC4807546

[28] Bergström A, Ehrenberg A, Eldh AC, Graham ID, Gustafsson K, Harvey G, et al. The use of the PARIHS framework in implementation research and practice—a citation analysis of the literature. Implement Sci. 2020;15(1):68. 32854718 10.1186/s13012-020-01003-0 PMC7450685

[29] Scott G, Hogden A, Taylor R, Mauldon E. Exploring the impact of employee engagement and patient safety. Int J Qual Health Care. 2022;34(3):1–8. 10.1093/intqhc/mzac059 PMC9384574 35899827

[30] Nilsen P, Seing I, Ericsson C, Birken SA, Schildmeijer K. Characteristics of successful changes in health care organizations: an interview study with physicians, registered nurses and assistant nurses. BMC Health Serv Res. 2020;20(1):147. 32106847 10.1186/s12913-020-4999-8 PMC7045403

[31] Ritchie MJ, Parker LE, Kirchner JE. From novice to expert: a qualitative study of implementation facilitation skills. Implement Sci Commun. 2020;1(1):25. 32885184 10.1186/s43058-020-00006-8 PMC7427882

[32] Gifford WA, Davies BL, Graham ID, Tourangeau A, Woodend AK, Lefebre N. Developing leadership capacity for guideline use: a pilot cluster randomized control trial. Worldviews Evid Based Nurs. 2013;10(1):51–65. 22647197 10.1111/j.1741-6787.2012.00254.x

[33] Ovretveit J. Improvement leaders: what do they and should they do? A summary of a review of research. Qual Saf Health Care. 2010;19(6):490–132. 21127110 10.1136/qshc.2010.041772

[34] Eldh AC, Hälleberg-Nyman M, Joelsson-Alm E, Wallin L. Facilitating facilitators to facilitate—some general comments on a strategy for knowledge implementation in health services. Front Health Serv. 2023;3:1112936. 37138952 10.3389/frhs.2023.1112936 PMC10149731

[35] Middleton R, Cardiff S, Manley K, Dewar B. Leadership relationships. In: Manley K, Wilson VJ, Oye C, editors. International practice development in health and social care. 2nd ed. Hoboken, NJ: Wiley; 2021. p. 159–72.

[36] Proctor E, Silmere H, Raghavan R, Hovmand P, Aarons G, Bunger A, et al. Outcomes for implementation research: conceptual distinctions, measurement challenges, and research agenda. Adm Policy Ment Health Ment Health Serv Res. 2011;38(2):65–76. 10.1007/s10488-010-0319-7 PMC3068522 20957426

[37] Hoffmann TC, Glasziou PP, Boutron I, Milne R, Perera R, Moher D, et al. Better reporting of interventions: Template for Intervention Description and Replication (TIDieR) checklist and guide. Br Med J. 2014;348:g1687. 24609605 10.1136/bmj.g1687

[38] Tong A, Sainsbury P, Craig J. Consolidated criteria for reporting qualitative research (COREQ): a 32-item checklist for interviews and focus groups. Int J Qual Health Care. 2007;19(6):349–57. 17872937 10.1093/intqhc/mzm042

[39] Savage C. Overcoming inertia in medical education navigating change with adaptative feflection. Karolinska Institutet. 2011. https://core.ac.uk/download/pdf/70339851.pdf. Accessed 10^th^ February 2024.

[40] Su WM, Osisek PJ. The revised Bloom’s taxonomy: implications for educating nurses. J Contin Educ Nurs. 2011;42(7):321–7. 21707023 10.3928/00220124-20110621-05

[41] Costea VA, Bäck A, Bergström A, Lundin A, Hasson H, Eriksson L. A longitudinal mixed methods evaluation of a facilitation training intervention to build implementation capacity. Front Health Serv. 2024;4:1–14. 10.3389/frhs.2024.1408801 PMC11427355 39347375

[42] Augustsson H, Costea V-A, Eriksson L, Hasson H, Bäck A, Åhström M, Bergström A. Building Implementation Capacity in health care and welfare through team training—study protocol of a longitudinal mixed-methods evaluation of the Building Implementation Capacity intervention. Implement Sci Commun. 2021;2(1):129. 34789320 10.1186/s43058-021-00233-7 PMC8596934

[43] Weiner BJ, Lewis CC, Stanick C, Powell BJ, Dorsey CN, Clary AS, et al. Psychometric assessment of three newly developed implementation outcome measures. Implement Sci. 2017;12(1):108. 28851459 10.1186/s13012-017-0635-3 PMC5576104

[44] Elo S, Kyngäs H. The qualitative content analysis process. J Adv Nurs. 2008;62(1):107–15. 18352969 10.1111/j.1365-2648.2007.04569.x

[45] World Medical Association (WMA). WMA declaration of Helsinki—ethical principles for medical research involving human subjects. 2024. https://www.wma.net/policies-post/wma-declaration-of-helsinki-ethical-principles-for-medical-research-involving-human-subjects/. Accessed 10^th^ January 2025.

[46] Britten N, Moore L, Lydahl D, Naldemirci O, Elam M, Wolf A. Elaboration of the Gothenburg model of person-centred care. Health Expect. 2017;20(3):407–18. 27193725 10.1111/hex.12468 PMC5433540

[47] Lood Q, Kirkevold M, Edvardsson D. Becoming part of an upwards spiral: meanings of being person-centred in nursing homes. Int J Older People Nurs. 2022;17(2):e12420. 34423910 10.1111/opn.12420

[48] McCance T, Dickson CAW, Daly L, Boomer CA, Brown D, Lynch B, et al. Implementing person-centred key performance indicators to strengthen leadership in community nursing: a feasibility study. J Nurs Manag. 2020;28(6):1443–52. 33448509 10.1111/jonm.13107

[49] Mosson R, Augustsson H, Bäck A, Åhström M, von Thiele SU, Richter A, et al. Building Implementation Capacity (BIC): a longitudinal mixed methods evaluation of a team intervention. BMC Health Serv Res. 2019;19(1):287. 31064362 10.1186/s12913-019-4086-1 PMC6505288

[50] Michie S, van Stralen MM, West R. The behaviour change wheel: a new method for characterising and designing behaviour change interventions. Implement Sci. 2011;6(1):42. 21513547 10.1186/1748-5908-6-42 PMC3096582

[51] Hardiman M, Dewing J. Using two models of workplace facilitation to create conditions for development of a person-centred culture: a participatory action research study. J Clin Nurs. 2019;28(15–16):2769–81. 31017323 10.1111/jocn.14897

[52] Mann K, Gordon J, MacLeod A. Reflection and reflective practice in health professions education: a systematic review. Adv Health Sci Educ. 2009;14(4):595–621. 10.1007/s10459-007-9090-2 18034364

[53] van Lieshout F, Cardiff S. Reflections on being and becoming a person-centred facilitator. Int Pract Dev J. 2015;5(Suppl):1–10.

[54] Lood Q, Carlström E, Klinga C, Barenfeld E. A collaborative endeavour to integrate leadership and person-centred ethics: a focus group study on experiences from developing and realising an educational programme to support the transition towards person-centred care. BMC Health Serv Res. 2024;24(1):395. 38553717 10.1186/s12913-024-10793-8 PMC10979622

[55] Geerligs L, Rankin NM, Shepherd HL, Butow P. Hospital-based interventions: a systematic review of staff-reported barriers and facilitators to implementation processes. Implement Sci. 2018;13(1):36. 29475440 10.1186/s13012-018-0726-9 PMC5824580

[56] van der Zijpp TJ, Niessen T, Eldh AC, Hawkes C, McMullan C, Mockford C, et al. A bridge over turbulent waters: illustrating the interaction between managerial leaders and facilitators when implementing research evidence. Worldviews Evid Based Nurs. 2016;13(1):25–31. 26788694 10.1111/wvn.12138

[57] Saarijärvi M, Bratt E-L. When face-to-face interviews are not possible: tips and tricks for video, telephone, online chat, and email interviews in qualitative research. Eur J Cardiovas Nurs. 2021;20(4):392–6. 10.1093/eurjcn/zvab038 PMC8135391 33893797

[58] Malterud K, Siersma VD, Guassora AD. Sample size in qualitative interview studies: guided by information power. Qual Health Res. 2016;26(13):1753–60. 26613970 10.1177/1049732315617444

