# Supplementary material for: Acceptability, appropriateness, and feasibility of an online facilitation training program designed to support the implementation of person-centered care in Swedish healthcare—a qualitative study
Source: Implement Sci Commun. 2025 May 30;6:68. doi: 10.1186/s43058-025-00752-7 (PMC12123759; doi:10.1186/s43058-025-00752-7)
Supplement: Supplementary file 3 — Supplementary Material 3. [file 43058_2025_752_MOESM3_ESM.docx]

**Interview guide**

Interview guide capturing the implementation outcomes, acceptability, appropriateness and feasibility of the FaciLitating Implementation of a Person-centered care (FLIP) training program for internal facilitators, managers and external facilitators.

**Questions to capture Acceptability** - *The perception among the study participants of the study whether FLIP was agreeable, palatable, or satisfactory*

- What do you think of FLIP?
- How were the parts that you attended: workshops, supervisions, work between workshops?

**Questions to capture Appropriateness -** *The perceived fit, relevance, or compatibility of FLIP for the healthcare units and the perceived fit of FLIP to support implementation of PCC by training healthcare staff to the asserted role as facilitators*

- How do you use what you learned during FLIP?
- How has the implementation model been used during the FLIP training?
- If you have worked with implementation and improvement work before: Are there differences in how you usually implement new methods compared to what you have learned during the training?

**Questions to capture Feasibility -** *The extent to which FLIP can be successfully used or carried out within their health care units*

- How has it worked out to participate in FLIP?
- How do you think the online format works?

How has the collaboration between managers and staff worked?

**Is there anything else you want to bring up that we haven't talked about today?**

For external facilitators some adjustments were made. To capture **Appropriateness** the question instead was: To what extent has FLIP been used at the healthcare units? To capture **Feasibility** the questions were: To what extent has FLIP been carried out within their health care units? How do you think the online format works? How was the collaboration between you and the facilitators? What do you think about attendance-participation?
